# Micro-Injection Moulding of Poly(vinylpyrrolidone-vinyl acetate) Binary and Ternary Amorphous Solid Dispersions

**DOI:** 10.3390/pharmaceutics11050240

**Published:** 2019-05-18

**Authors:** Romina Pezzoli, Michael Hopkins Jnr, Guillaume Direur, Noel Gately, John G. Lyons, Clement L. Higginbotham

**Affiliations:** 1 Applied Polymer Technologies, Athlone Institute of Technology, Dublin Road, Athlone, N37 HD68 Co. Westmeath, Ireland; rpezzoli@ait.ie (R.P.); Mhopkins@ait.ie (M.H.J.); n.gately@ait.ie (N.G.); 2 Synthesis and Solid State Pharmaceutical Centre (SSPC), Dublin Road, Athlone, N37 HD68 Co. Westmeath, Ireland; 3 Materials Research Institute, Athlone Institute of Technology, Dublin Road, Athlone, N37 HD68 Co. Westmeath, Ireland; Guillaume.direur@gmail.com; 4 Faculty of Engineering and Informatics, Athlone Institute of Technology, Dublin Road, Athlone, N37 HD68 Co. Westmeath, Ireland; slyons@ait.ie

**Keywords:** amorphous solid dispersions, class II drugs, continuous processing, micro-injection moulding, indomethacin, poly(vinylpyrrolidone-vinyl acetate)

## Abstract

Micro-injection moulding (µIM) was used for the production of enteric tablets of plasticised and unplasticised solid dispersions of poly(vinylpyrrolidone-vinyl acetate) (PVPVA), and the effect of the mechanical and thermal treatment on the properties of the dispersions was investigated. The physical state of the systems showed to be unaltered by the µIM step, maintaining the drug in the amorphous state. The dissolution profile of the tablets showed a slower dissolution rate due to the lower surface to volume ratio compared to the extruded strands. The lack of solubility of the doses in the acidic medium as a consequence of the acidity of indomethacin (IND) was observed. However, in neutral pH the drug dissolution showed slower rates without affecting the dissolution extent, showing a potential application for the development of controlled release doses. Overall, the production of tablets of amorphous solid dispersions (ASD), coupling hot-melt extrusion (HME) and µIM, proved to be a successful approach towards a continuous automated manufacturing process to improve the aqueous solubility of poorly water-soluble drugs.

## 1. Introduction

The research of amorphous solid dispersions (ASD) to improve the solubility of class II active pharmaceutical ingredients (APIs) points out hot-melt extrusion (HME) as one of the most appealing techniques for their manufacture due to its continuity and solvent-free nature [1,2,3]. Traditionally, the process to make oral tablets consists of two steps: granulation and subsequent direct compression. Accordingly, solid dispersions produced using HME would have to be pelletised, pulverised and finally pressed into tablets. The requirement of those steps defeats the advantage of continuous processing, one of the most attractive characteristics of integrating HME in the manufacturing of pharmaceuticals. In order to overcome this barrier and move towards a more continuous process, injection moulding (IM) has been identified as a technique with great potential for the manufacture of drug delivery systems of defined shapes and dimensions [4,5,6,7]. 

Combining HME and IM represents a step forward in the development of a continuous automated process for the manufacture of amorphous pharmaceutical doses with high potential for scaling up to mass production. The FDA has outlined the benefits in the implementation of continuous manufacturing of pharmaceuticals and the related potential cost reductions through reducing steps by integrating processes, increasing safety by minimising manual handling, reducing of processing times, the increment of efficiency, on-line monitoring for real-time quality assurance and consistent quality [8].

However, several questions arise when considering the IM of ASD, for example, the processability of pharmaceutical grade polymers, the dissolution mechanism of these highly dense doses and the impact of the thermal and mechanic treatments on the stability of the drug’s amorphous state [9,10]. In order to broaden the understanding of the subject and to propose a continuous method for the manufacture of ASD, this investigation focuses on the preparation and characterisation of oral doses manufactured using micro-injection moulding (μIM).

μIM was developed in the 1990s to target the needs of the manufacture of micro-structured parts. μIM does not consist of simply scaling down the conventional IM as specific features with different behaviours need to be addressed: accurate metering and dosing, small shot size, high injection rate and short response time [7].

During the μIM process, the polymer flows through small size runners and gates at high speed and pressure. This generates high shears, which, in turn, can cause the degradation of the material. To avoid this counter effect, together with the reduction of waste, two main concepts were developed in the design of this technology [11].

(a) The reduction of the size of the plasticisation unit due to the little amount of material needed for the fabrication of microparts. In the case of systems with barrel and screw, the dimensions are reduced down to 20 mm in diameter with the lower limit close to 12 mm, due to the standard size of plastics pellets.

(b) Separation of the plasticisation and injection units. Separated plasticisation units offer more efficiency and homogeneity of the melt. The injection unit pushes the melted material into the cavities by the use of a small plunger (few millimetres in size) that provides better control of the amount of material injected for the same displacement.

Considering the knowledge developed on the processing and behaviour of poly(vinylpyrrolidone-vinyl acetate) (PVPVA) and indomethacin (IND) based solid dispersions, this system was selected as model compounds for the design of moulded tablets [12,13]. PVPVA-IND ASD were produced using twin-screw HME. The solid dispersions were then shaped in the form of tablets using μIM technique. The processability of the amorphous PVPVA represented one of the main and novel challenges due to its high melt viscosity and fragile mechanical behaviour. For this aim, the inclusion of polyethylene oxide (PEO) as a plasticiser was evaluated as it was previously proved that this system reduces the system viscosity without affecting its solid stability [13]. It is also of great interest to this investigation to evaluate the impact of the thermal treatment associated with the µIM process over the phase behaviour of the moulded tablets.

## 2. Materials and Methods

### 2.1. Materials

Copolymer PVPVA in a ratio of 6:4 by mass, was purchased from BTC Chemical Distribution (Burgbernheim, Germany). PEO, molecular weight 600,000 was sourced by Sigma Aldrich (Arklow, Ireland). Poorly water-soluble IND, 1-(4-Chlorobenzoyl)-5-methoxy-2-methyl-3-indoleacetic acid, was purchased from Tokyo Chemical Industry UK Ltd (Oxford, UK). 

### 2.2. Methods

#### 2.2.1. Differential Scanning Calorimetry

Differential scanning calorimetry (DSC) analysis was carried out using a Perkin Elmer Pyris 6 DSC (Perkin Elmer, Beaconsfield, UK). Samples between 4 and 6 mg were accurately measured and placed into open aluminium pans. The samples were heated from room temperature to 200 °C using a heating rate of 10 °C/min. Calorimetry scans were performed under a nitrogen atmosphere with a steady flow of 20 mL/min to prevent oxidation. Samples were tested in triplicates.

#### 2.2.2. Hot-Melt Extrusion

Melt compounding was carried out on a bench-top Prism™ twin-screw extruder with 16 mm diameter screws and a 25:1 length to diameter ratio (Thermo Electron Corporation, Staffordshire, UK). The co-rotating intermeshing screws were configured with the inclusion of individual kneading paddles with a design of 30°, 60° and 90° twist angles to generate a high mixing effect on the dispersions. The temperature profile from the feeding zone to die was 85 °C/130 °C/150 °C/160 °C for the binary dispersions. With the inclusion of the plasticiser, the temperature profile was reduced by 10 °C. The physical mixtures were fed into the extruder using an automatic feeder at a rate of 13 ± 1 g/min. The composition of the formulations is presented in Table 1.

#### 2.2.3. Micro-Injection Moulding

The µIM machine used in this study is the Babyplast 6/10P (Cronoplast, S.L., Barcelona, Spain) with a plasticising unit composed by a plasticisation chamber. Extruded dispersions of PVPVA and IND with and without PEO, were loaded into the hopper and transferred into the plasticising chamber, heated at a controlled temperature to ensure the melt and homogenisation of the material. The temperature was set up above the melting temperature of the drug due to the short resident times and lack of shear from the screw. The materials were pushed into the injection chamber by a plunger where the melt was injected into the mould at a determined shot size to fill the cavities. The temperatures of the injection chamber and nozzle were set separately, with the second lower than the first one to avoid melt dripping. Two different pressures were applied. The first injection pressure pushed the melt into the moulding tool to fill up the cavities and compress the material. The second pressure or holding pressure was applied to maintain the compression in the cavity until the solidification of the gate. The mould was kept close for 20 seconds to allow the parts to cool down and solidify. The total cycle time was 33 s. The processing conditions for the binary and ternary solid dispersions are detailed in Table 2.

#### 2.2.4. Morphology Analysis

The morphology of the injection moulded tablets was evaluated using a scanning electron microscope (SEM). Samples were sputter coated with gold using a Baltec SCD 005 sputter coater (BAL-TEC, Schalksmühle, Germany) and placed on the sample holder before testing. A Mira FE SEM (Tescan, Oxford Instruments, Cambridge, UK) was used in high vacuum mode by scanning a beam of high energy electrons across the surface of the samples. The setup parameters used were a resolution of 10 μm at 10 kV, magnification ranges between 500 x and 2 kx.

#### 2.2.5. Attenuated Total Reflectance Fourier Transformation Infrared Spectroscopy (ATR–FTIR)

Samples were analysed using a Perkin Elmer Spectrum One (Perkin Elmer, Beaconsfield, UK) fitted with a universal ATR sampling accessory. Samples were tested at room temperature, and spectra were recorded over the 4000–650 cm^−1^ wavelength range using ten scans per cycle and under a compression force of 80 N.

#### 2.2.6. Dissolution Studies

In vitro drug dissolution studies were performed using a Distek dissolution apparatus 1 using baskets according to the USP 711 guideline (Distek Inc., North Brunswick, NJ, USA). Tests were carried out in 900 mL of dissolution medium at 37.0 ± 0.5 °C and a stirring speed of 50 rpm. Samples of 2 mL were taken from the dissolution vessels at adequate periods of time and replaced with the same volume of the medium. Dissolution studies were performed in 1.2 pH hydrochloric acid buffer and 6.8 pH phosphate buffer media to address the pH-sensitivity of IND and to simulate the digestive tract. The buffer solutions were prepared according to the directions of the U.S. Pharmacopeia USP29 [14]. The reagents to prepare the buffer solutions, hydrochloric acid at 37% (HCl), potassium chloride (KCl), monobasic potassium phosphate (KH_2_PO_4_) and sodium hydroxide (NaOH) were purchased from Sigma Aldrich (Arklow, Ireland).

#### 2.2.7. High-Performance Liquid Chromatography

The concentration of IND was determined using high-performance liquid chromatography (HPLC). HPLC analysis was carried out using a system consisting of a Waters Alliance e2695 separations module (Waters Chromatography Ireland Limited, Dublin, Ireland) combined with a Waters 2487 dual λ absorbance detector. A 150 mm × 4.6 mm Thermo Scientific ODS Hypersil column with a particle size of 5 μm (Fisher Scientific Ireland Ltd, Dublin, Ireland) was used for separation and quantitation of the IND content. The mobile phase was a solution of 0.01 M monobasic sodium phosphate and 0.01 M dibasic sodium phosphate in HPLC grade acetonitrile (Romil) and distilled water 1:1. Both salts were obtained from Sigma Aldrich. The mobile phase was filtered through 0.20 μm nylon filters (Agilent Technologies Ireland, Cork, Ireland) and degassed under vacuum. A flow rate of 1 mL/min was maintained during the procedure, the detector was set at 254 nm, and the samples injection volume was 20 µL. A filtered solution of acetonitrile and water 1:1 was used to wash the needle between injections. Before testing, samples were diluted in the mobile phase to inhibit the precipitation of the drug.

## 3. Results and Discussion

### 3.1. Design of the Tablet Injection Mould

**Figure 1:**
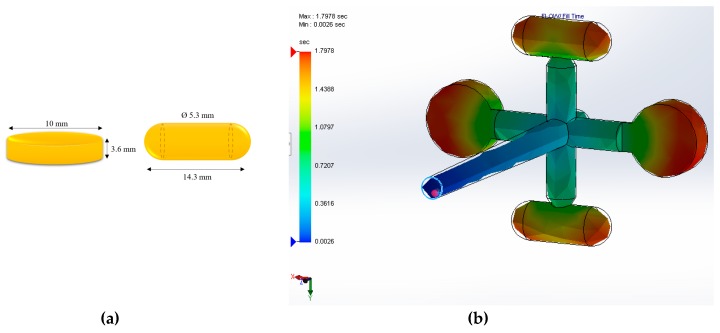
(**a**) Dimensions of the injection mould cavities in the shape of tablet and capsule, (**b**) fill time profile of the tablet injection mould.

The cavity in the µIM tool was designed using the software Solidworks® Plastics. The design consisted of four cavities, two for capsules and two for round tables. The dimensions of the parts cavities are presented in Figure 1a. The size of the cavities was designed to match the therapeutic dose of IND of 25 mg and 50 mg with the solid dispersion formulations presented in Table 1. In this body of work, the study of the µIM of solid dispersions was limited to the cylindrical tablets. 

The simulation of the fill time of the cavities was performed in Solidworks® Plastics as part of the designing process and to generate processing information to use as a guideline to start the manufacturing process at the machine. In Figure 1b, the fill time plot displays the profile of the plastic melt as it flows through the mould cavity during the filling stage of the µIM process. The blue region corresponds to the start of the flow front and the red to the end of fill when the flow has stopped. The resulting filling time was 1.79 s, which is in good correlation with the filling time reached during the processing of the tablets (2 s as detailed in Table 2).

### 3.2. Micro-Injection Moulding of Tablets

**Figure 2:**
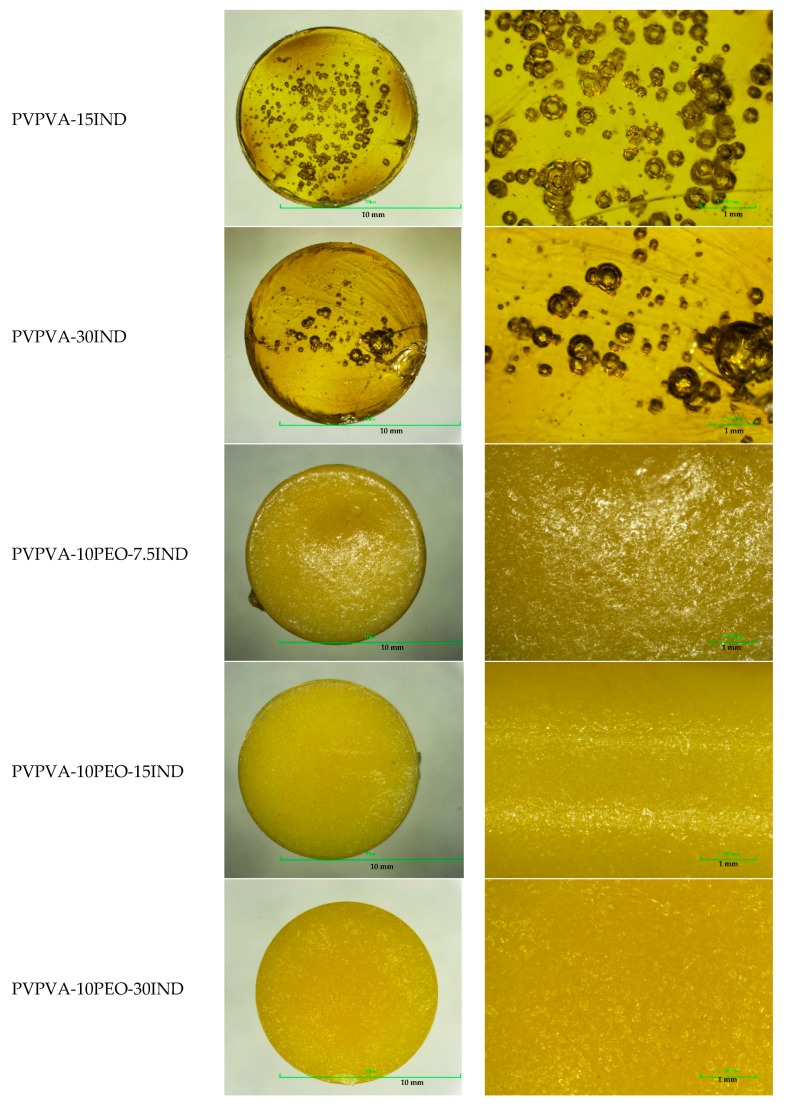
Physical aspect of moulded solid dispersions tablets.

Due to the high melt viscosity of the materials, tablets of the binary solid dispersions containing 7.5% IND could not be moulded using the standard processing equipment available without degrading the material through excess shear or heating. The physical aspect of the moulded tablets is presented in Figure 2. It is apparent for the binary dispersions, that the tablets present numerous voids in their centre, probably due to the high viscosity and quick solidification rate of the material. Due to the high glass transition temperature of PVPVA (108.3 ± 0.5 °C), which is the main component of all the systems evaluated, the glass transition temperatures of all the systems are relatively high (in between 75 °C and 100 °C). Considering the cooling process of the tablets during the µIM cycle, the transition from rubbery to vitreous or “freezing” of the material, will occur rather quickly, decreasing the effect of the holding pressure to compact the dose. Additionally, the binary tablets turned out to be extremely brittle and, due to residual stress after injection and the stress applied at the moment of their ejection from the moulding tool, they tend to crack and break. This is shown in Figure 2 where fracture lines are noticeable. Ternary dispersion tablets presented a different aspect and increased toughness due to the presence of the plasticiser. Their opaque colour is due to the semi-crystalline nature of the PEO.

The weight of the moulded tablets was recorded to evaluate the consistency of the µIM process over time. Weight control is an economical, easy way to identify variations during the processing as it is affected by processing variables such as injection temperature, mould temperature, injection pressure and holding pressure [15,16,17]. The results of the weight uniformity are presented in Table 3 showing small standard deviations, lower than 3% of the weight of the tablet.

The uniformity of content of the moulded tablets was evaluated through the measuring of IND content using HPLC and results are presented in Table 3. For all the formulations analysed, IND showed a single peak in the chromatographs at an elution time of 1.78 minutes, confirming that the moulding process did not promote degradation of the API. Quantitative results showed that the IND content was consistent with a difference lower than ± 10% between the nominal content and concentration obtained experimentally. The tablets were successfully produced achieving a consistent and uniform drug content. This result matches the USP guideline for IND tablets where a tolerance in the drug content range from 100 ± 10% from the labelled amount is allowed [18].

### 3.3. Morphology Analysis

**Figure 3:**
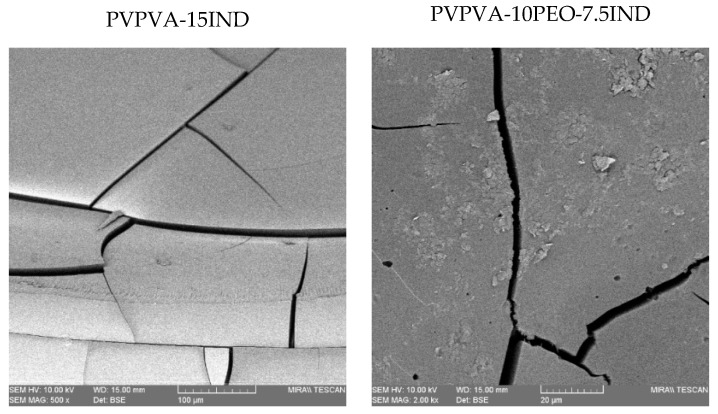

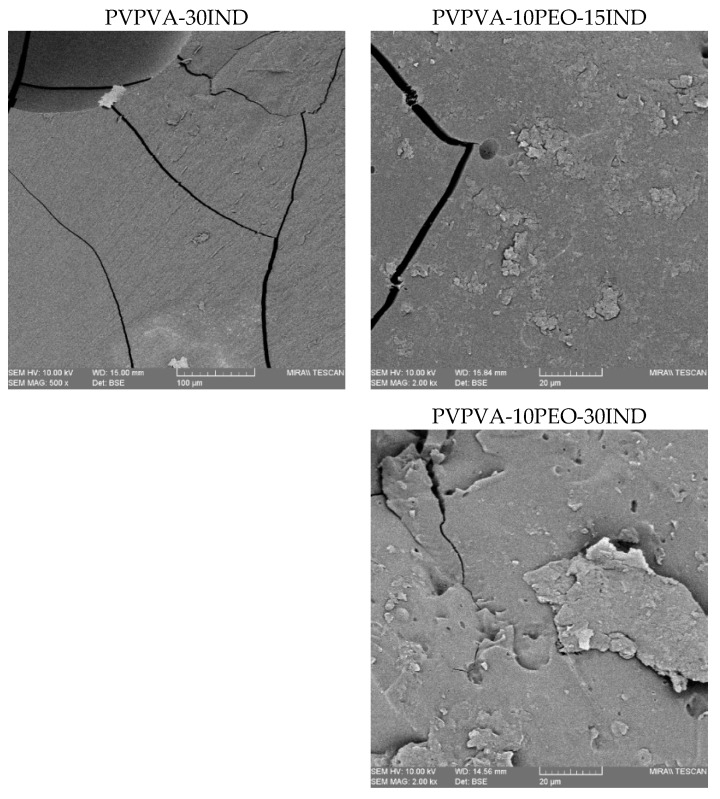
SEM images of μIM tablets.

Tablets were analysed using SEM to explore their morphology and the results are presented in Figure 3. It can be observed that the binary system presented a uniform phase and drug crystals were not detected. On the contrary, the images of the ternary system showed a second phase attributed to the presence of the semi-crystalline plasticiser. These results are in agreement with the thermal characterisation of the extrudates investigated in a previous work where the ternary dispersions coexisted as a mixture of an amorphous homogenous phase with dispersed PEO crystals [13]. The SEM findings suggest that the morphology of the μIM tablets are similar to the extrudates. This will be confirmed by the thermal characterisation of the doses. 

### 3.4. Thermal Analysis

**Figure 4:**
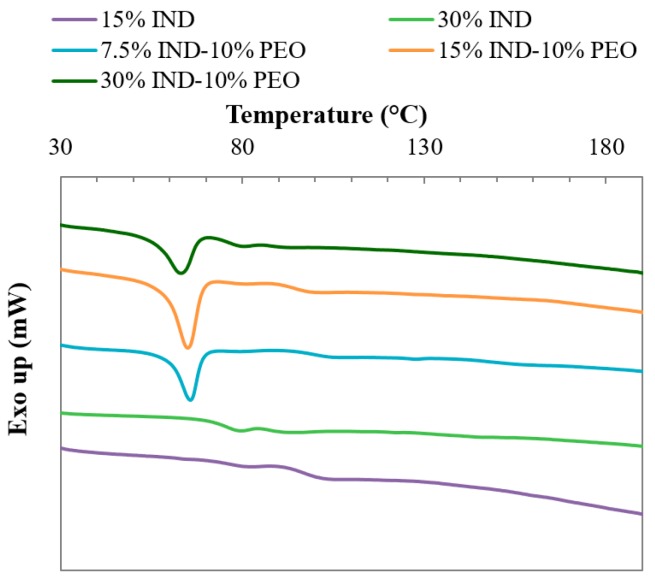
DSC thermograms of binary and ternary injection moulded solid dispersions of PVPVA-IND and PEO.

DSC analysis was performed on the tablets to investigate the physical state of the dispersions after the moulding process. The thermograms of the binary and ternary moulded dispersions are presented in Figure 4 and the values of the thermal transitions are detailed in Table 4. Both of the binary dispersions analysed showed to be amorphous as no endothermic peaks related to the melting of IND were detected (*T*_m_ = 164 ± 1 °C). A glass transition temperature of 95.8 ± 0.7 °C and 74 ± 1 °C for the doses with 15% and 30% of the drug were obtained confirming the known plasticising effect of the drug in the polymer. The thermograms of the ternary dispersions showed the melting peak of the crystalline fraction of PEO at temperatures between 65.9 and 62.5 °C. Also, the ternary systems exhibited one glass transition temperature that decreases with the content of IND. It is suspected that the system is composed of an amorphous miscible matrix composed of PVPVA-IND with dispersed crystalline PEO. These findings are in agreement with the SEM results that showed a crystalline phase dispersed in the amorphous matrix.

The results obtained for all the moulded dispersions investigated are similar to the thermal analysis of the extruded dispersion analysed in a previous investigation confirming that the thermal treatment and high shear applied during the moulding process did not alter the solid state of the systems [13,19,20].

### 3.5. Investigation of Intermolecular Interactions

**Figure 5:**
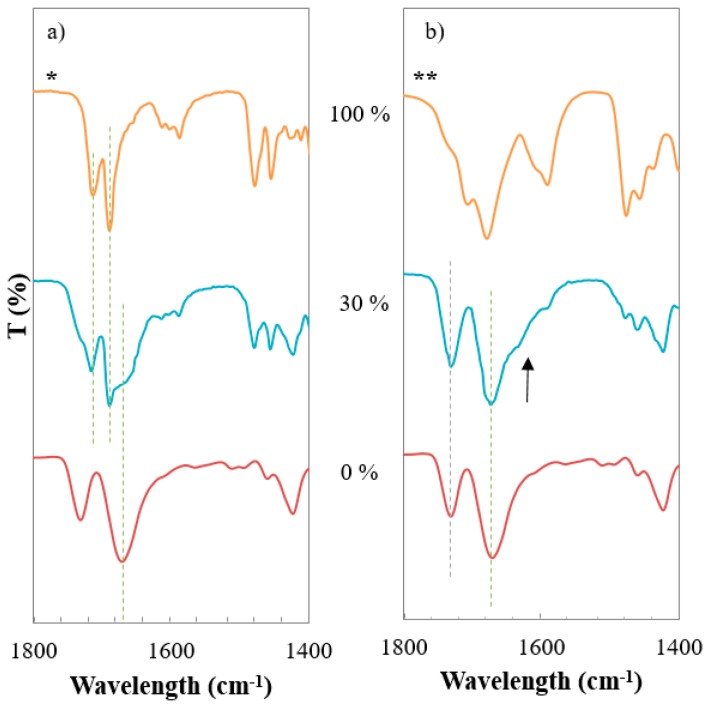
Attenuated total reflectance Fourier transformation infrared spectroscopy (ATR-FTIR) spectra of (**a**) physical mixtures, (**b**) solid dispersions of PVPVA-IND. Numbers indicate the content of IND, * ϒ-form IND, ** amorphous IND. The lines mark the position of the carbonyl bands and the arrow indicates the appearance of a new band.

The evaluation of the nature and extent of the molecular interactions between the polymer and the drug in ASD is a fundamental factor to understand the particularities of each system. The possible occurrence of intermolecular interactions between the carriers and the drug was investigated using FTIR (Figure 5). PVPVA is a non-ionic copolymer that contains two carbonyl groups (C=O) in its structure. Bands located at 1731 cm^−1^ and 1671 cm^−1^ correspond to the vibration of the carbonyl groups of the acetate group and pyrrolidone ring, respectively. These carbonyl functions are strong hydrogen bond acceptors, with the first stronger than the second. The pyrrolidone group is substantially more basic than the acetate group and in consequence, it can form stronger interactions with proton donors groups [21]. However, this material does not have proton donors in its structure, and as a result, despite the capability of the hydrogen bonding formation of the two carbonyl groups, no interactions occur in its pure state [22].

Two forms of IND were analysed using FTIR, the stable crystalline γ-form and the amorphous form obtained by quenching the material from the melt. IND contains two carbonyl groups: benzoyl and acid. In the spectrum of γ-IND (Figure 5a), the band corresponding to the stretching of the benzoyl carbonyl group appeared at 1690 cm^−1^, and a shift to lower wavelengths was observed for the amorphous IND. This observation is linked with a reduction of the conformational restrictions. The spectrum of γ-IND shows a band at 1714 cm^−1^ assigned to the acid carbonyl stretch, stretch that has been reported as evidence of the formation of cyclic dimers. In the spectrum of the amorphous IND, the same band is shifted to 1707 cm^−1^, and a shoulder becomes apparent at 1732 cm^−1^, a vibration assigned to the stretch of the non-hydrogen bonded carbonyl groups. This observation suggests that in the amorphous state, IND is bonding as dimers and a small proportion of molecules are bonding to form a chain with unbounded carbonyl groups at the end of these chains [23]. 

By analysing the physical mixtures’ spectra (Figure 5a) it can be observed that, despite the presence of proton acceptor groups in the polymer and the proton donor groups of IND, no intermolecular interactions were detected. The mixtures’ spectra correspond to the superposition of the pure materials spectra with no evidence of new bands or change in the wavelength of the existing ones. The lack of intermolecular interactions between the materials may be due to the persistence of hetero-interactions formed between IND molecules. The spectra of the extruded solid dispersions containing 30% of IND revealed the appearance of a new shoulder in the pyrrolidone’s carbonyl peak at 1635 cm^−1^ (highlighted with an arrow in Figure 5b) that may suggest the occurrence of new intermolecular interactions. Yuan et al. reported, that in solid dispersions of PVPVA and IND, the presence of the polymer interfered with the formation of the IND carboxylic acid dimers and new H-bonds between the drug and the polymer were favoured [24]. This same tendency was also reported in mixtures of PVP and IND, suggesting that PVP could interfere with the crystallisation kinetics of the API by preventing its self-association [23].

**Figure 6:**
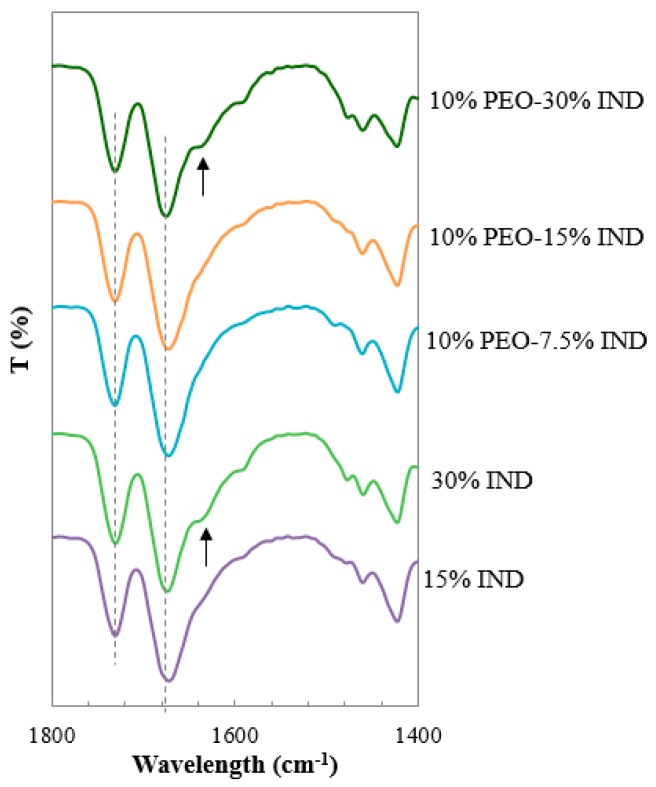
ATR-FTIR spectra of binary and ternary injection moulded solid dispersions PVPVA-IND and PEO. The lines mark the position of the carbonyl bands and the arrows indicate the appearance of new bands.

Intermolecular interactions have been seen to intensify after promoting intensive mixing via HME as it was demonstrated in Figure 5, showing that the level of mixing between the components has an impact on the intensity of the interactions. Considering the thermal and mechanical treatment applied to the samples during the µIM process, the evaluation of possible changes in the intermolecular interactions between the materials was explored. Results obtained from the FTIR analysis of the enteric tablets of binary and ternary solid dispersions are presented in Figure 6. 

The systems with 7.5% and 15% content of IND presented no evidence of interactions on their carbonyl bands. Only formulations with higher contents of the drug, 30%, exhibited a shoulder on the pyrrolidone carbonyl band at 1638 cm^−1^. This new band, located at the same wavelength for the extruded dispersion of PVPVA-IND is attributed to the formation of hydrogen bonding between the pyrrolidone carbonyl and IND. No difference between the binary and ternary dispersions were detected confirming that the presence of the plasticiser PEO has no effect on the interactions between PVPVA and the drug.

### 3.6. Dissolution Studies

**Figure 7:**
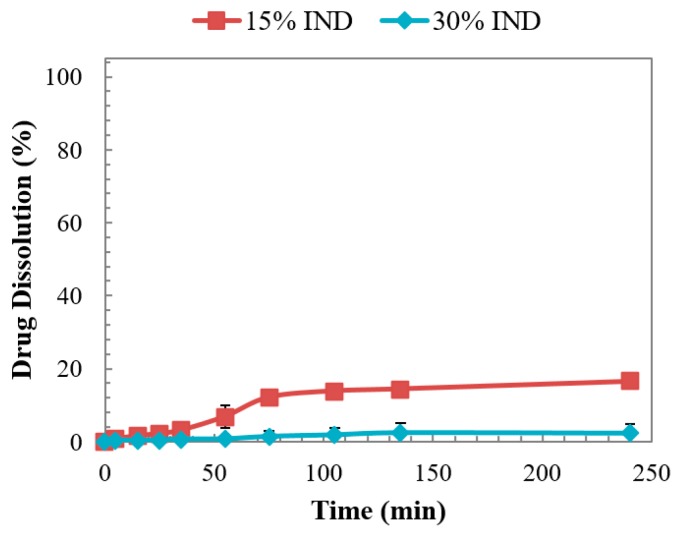
Dissolution of injection moulded tablets of PVPVA-IND solid dispersions in pH 1.2 dissolution medium.

Investigation of the dissolution behaviour of the moulded tablets were evaluated in acidic pH to simulate the gastric environment and study the pH-dependent solubility behaviour of IND. The concentration of IND was determined using HPLC and the results obtained for the binary dispersions are presented in Figure 7.

**Figure 8:**
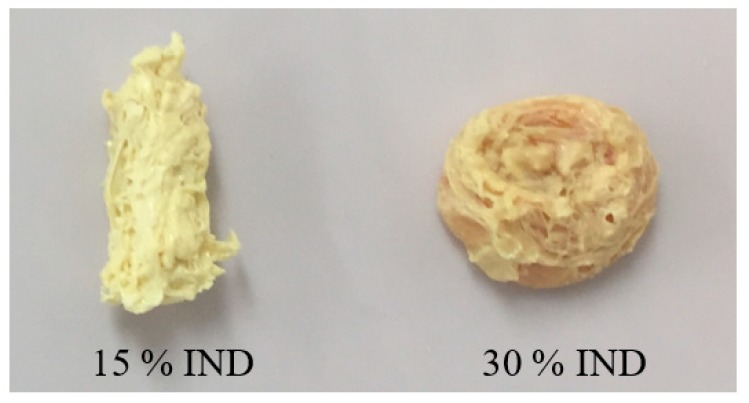
The physical appearance of post-dissolution injection moulded tablets of PVPVA-IND solid dispersions in pH 1.2 dissolution medium.

Doses failed to disintegrate due to the acidic nature of the drug and the formation of a hydrophobic solid was observed. This result has been reported by other researchers and by the authors in a previous work where extruded solid dispersions of PVPVA and IND, with drug contents higher than 10% do not disintegrate due to the pH controlled dissolution behaviour [13,25]. The physical aspect of the post-dissolution non-dissolved tablets is presented in Figure 8, showing the evident deformation of the dose with 15% of IND due to its hydration against the rounded solid obtained from the dose with 30% of the drug. This deformation may have contributed to the drug release observed for the enteric tablet.

**Figure 9:**
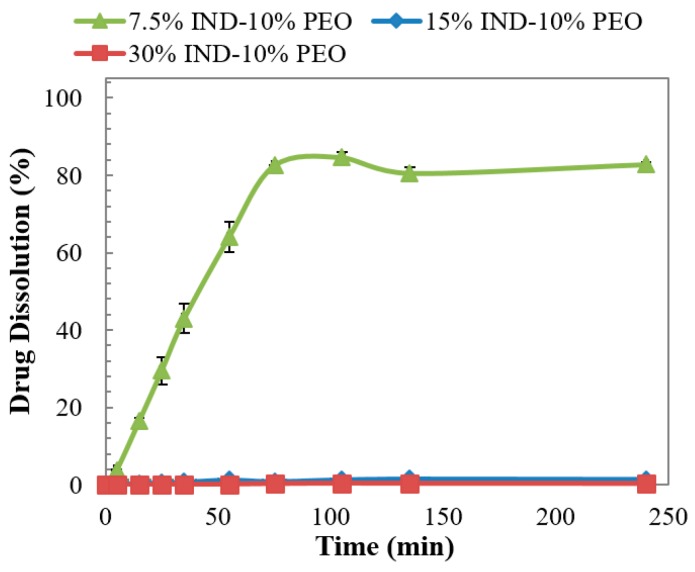
Dissolution of injection moulded tablets of PVPVA-PEO-IND solid dispersions in pH 1.2 dissolution medium.

Results of dissolution studies of the moulded enteric tablets of ternary solid dispersions in the acidic medium are presented in Figure 9. The dissolution rate of the dose with the lower content of the drug was decreased reaching a drug dissolution higher than 80% after 75 min compared to the extruded dispersion which showed an instant release (higher than 80% after 20 min) [13]. This decrease in the dissolution rate is due to the lower surface area in contact with the dissolution medium when compared to the HME strands. Additionally, due to the packing during moulding, the doses are highly dense, which may retard the drug diffusion process [26]. 

**Figure 10:**
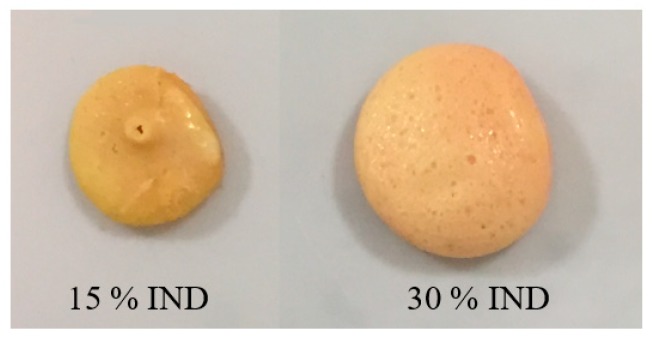
The physical appearance of post-dissolution injection moulded tablets of PVPVA-PEO-IND solid dispersions in pH 1.2 dissolution medium.

Similarly to the systems previously evaluated, doses with a higher content of IND failed to dissolve in the acidic pH. The physical appearance of the post-dissolution tablets is presented in Figure 10. Unlike the previous case, the samples retained their physical shape showing a smoother surface than that observed for the binary dispersion tablets. The difference in the appearance is attributed to the presence of PEO which, due to its relatively high molecular weight and partially crystalline state, is related to slower disintegration rates, preventing the deformation by the superficial absorption of the medium.

**Figure 11:**
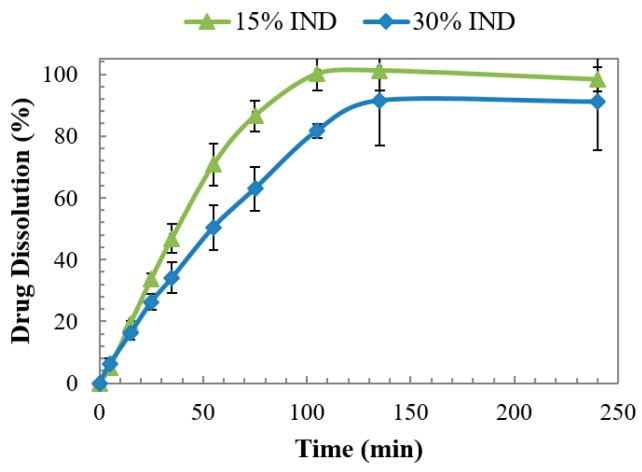
Dissolution of injection moulded tablets of PVPVA-IND solid dispersions in pH 6.8 dissolution medium.

The dissolution behaviour of the binary dispersions tablets was also evaluated in a neutral medium of pH 6.8 to simulate the intestinal environment and investigate the dissolution of the acidic tablets in a favourable medium. Results are presented in Figure 11 where a complete drug release (higher than 90%) for both doses is observed. The dissolution rate proved to be dependent on the IND content, exhibiting a slower release with the increase of the drug loading. Analysing the dissolution profile of the tablet with 15% IND and comparing it with the equivalent extruded formulation, there is a steady reduction of the dissolution rate (IM tablet 16% after 15 minutes vs. extruded dose >90% after 15 min). The difference in rate is solely attributed to the change in the geometry and unit size of the doses as the surface area is proportional to the dissolution rate [27]. That is the case of the characterisation of extruded and injection moulded doses of Soluplus and fenofibrate where the surface-to-volume ratio of the doses determined the dissolution profile. This is due to changes of the gel layer which, for doses with an increased surface to volume ratio, the drug travelled shortened diffusion lengths [19].

**Figure 12:**
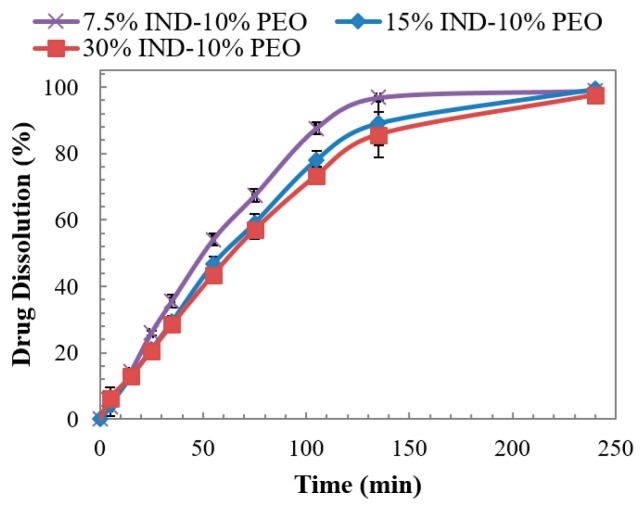
Dissolution of injection moulded tablets of PVPVA-PEO-IND solid dispersions in pH 6.8 dissolution medium.

The dissolution profiles of the ternary injection moulded tablets in neutral pH are presented in Figure 12. All three formulations showed a complete drug release with slower dissolution rates than the binary tablets from Figure 11. This decrease in the dissolution rate is due to the presence of semi-crystalline PEO, as previously explained. Also, the dissolution rate showed to be slightly affected by the drug content, showing a difference between the faster dissolution of tablets with 7.5% against the other two formulations. This dependency of the rate with the drug content is a repetitive pattern related to the hydrophobic nature of the dose. However, the difference in the dissolution rate with the IND content showed to be more pronounced for the binary system than for the ternary system. This suggests that the presence of PEO and its dissolution, controlled by its high molecular weight and semi-crystalline state, modulated the dissolution behaviour [28,29]. As a consequence, in a neutral pH where the solubility of the acidic drug is higher, PEO dominated the dissolution mechanism.

## 4. Conclusions

The use of µIM for the production of enteric tablets for oral dose applications was investigated through the use of unplasticised and plasticised solid dispersions of PVPVA-IND and PEO. Solid dispersions obtained using HME were processed using a µIM machine to obtain tablet shaped parts. Thermal analysis of the moulded tablets showed that the mechanical and thermal treatment applied during the µIM process did not promote changes in the physical state of the doses. IND remained in the amorphous state and the PEO fraction persevered in its dispersed semi-crystalline morphology. 

The dissolution behaviour of the tablets, similarly to the extruded doses, showed a strong dependency with the pH due to the acidic nature of IND. Nevertheless, the tablets showed a remarkable decrease in the dissolution rate due to the reduced surface to volume ratio when compared to the extruded doses. Within the dissolution profile of the tablets, leaving the geometry factor aside, the dissolution rate was shown to be dependent on the IND content and that effect got intensified with the presence of the plasticiser, showing a potential strategy for improving the processability and modulating the dissolution profiles. The results obtained offer a solid foundation for the development of oral doses using an automatized, stable and more continuous manufacturing process and achieving final products with improved solubility and regulated dissolution behaviours.

## Figures and Tables

**Table 1:** Drug/carrier percentages of the HME processed formulations.

Formulation	Content % (*w*/*w*)		
	PVPVA	PEO	IND
PVPVA-7.5IND	92.5	-	7.5
PVPVA-15IND	85.0	-	15.0
PVPVA-30IND	70.0	-	30.0
PVPVA-10PEO-7.5IND	82.5	10.0	7.5
PVPVA-10PEO-15IND	75.0	10.0	15.0
PVPVA-10PEO-30IND	60.0	10.0	30.0

**Table 2:** Processing conditions for the µIM of binary and ternary solid dispersions of poly(vinylpyrrolidone-vinyl acetate)- indomethacin (PVPVA-IND) and polyethylene oxide (PEO).

Variable	Binary SD	Ternary SD
Plasticisation temperature (°C)	190	170
Injection chamber temperature (°C)	160	160
Shot size (mm)	15	15
Injection pressure (Bar)	60	60
Injection time (s)	2	2
Holding pressure (Bar)	20	20
Holding time (s)	6	6
Mould temperature (°C)	35	35

**Table 3:** Uniformity of weight and IND content of tablets fabricated using µIM.

Formulation	Tablet Weight (mg)	IND (%)
PVPVA-15IND	349 ± 8	13.7 ± 0.3
PVPVA-30IND	360 ± 6	26.9 ± 0.9
PVPVA-10PEO-7.5IND	349 ± 2	7.20 ± 0.03
PVPVA-10PEO-15IND	352 ± 2	14.9 ± 0.2
PVPVA-10PEO-30IND	357 ± 7	28 ± 1

**Table 4:** Glass transition temperature of binary and ternary injection moulded solid dispersions of PVPVA-IND and PEO.

Formulation	*T*_g_ (°C)	*T*_m_ (°C)	Δ*H*_m_ (J/g)
PVPVA-15IND	95.8 ± 0.7	-	-
PVPVA-30IND	74 ± 1	-	-
PVPVA-10PEO-7.5IND	99.2 ± 0.5	65.9 ± 0.3	8.7 ± 0.4
PVPVA-10PEO-15IND	94.6 ± 0.2	65.3 ± 0.3	9 ± 1
PVPVA-10PEO-30IND	76 ± 2	62.5 ± 0.6	8 ± 1
